# Comparative Assessment of Percutaneous Left-Atrial Appendage Occlusion (LAAO) Devices—A Single Center Cohort Study

**DOI:** 10.3390/jcdd11060158

**Published:** 2024-05-21

**Authors:** Elham Kayvanpour, Max Kothe, Ziya Kaya, Sven Pleger, Norbert Frey, Benjamin Meder, Farbod Sedaghat-Hamedani

**Affiliations:** 1 Department of Internal Medicine III, Heidelberg University, 69120 Heidelberg, Germany; elham.kayvanpour@med.uni-heidelberg.de (E.K.);; 2 DZHK (German Centre for Cardiovascular Research), 69120 Heidelberg, Germany; 3 Department of Genetics, Stanford Genome Technology Center, Stanford University School of Medicine, Stanford, CA 94305, USA

**Keywords:** left atrial appendage closure, atrial fibrillation, oral anticoagulation

## Abstract

Background: Percutaneous left-atrial appendage closure (LAAC) is an established method for preventing strokes in patients with atrial fibrillation, offering an alternative to oral anticoagulation. Various occluder devices have been developed to cater to individual anatomical needs and ensure a safe and effective procedure. In this retrospective, monocentric cohort study, we compare different LAAO devices with respect to clinical outcomes, LAA sealing properties, and device-related complications. Methods: We conducted a retrospective analysis of 270 patients who underwent percutaneous LAA closure in our center between 2009 and 2023. Patient data were extracted from medical records, including gender, age at implantation, indication, device type and size, laboratory values, LAA anatomy, periprocedural complications, ECG parameters, transthoracic and transesophageal echocardiography parameters (TTE and TEE), as well as medication at discharge. Moreover, fluoroscopy time and implantation duration, as well as post-implantation clinical events up to 1 year, were collected. Endpoints were bleeding events, recurrent stroke, thrombi on devices, and death. Results: The implanted devices were the Watchman 2.5, Watchman FLX, Amplatzer Cardiac Plug (ACP), and Amulet. The procedural success rate was 95.7% (*n* = 265), with cactus anatomy posing the most challenges across all devices. The mean patient age was 75.5 ± 7.7 years, with 64.5% being male. The median CHA2DS2-VASc score was 4.8 ± 1.5 and the median HAS-BLED score was 3.8 ± 1.0. Indications for LAA closure included past bleeding events and elevated bleeding risk. Periprocedural complications were most commonly bleeding at the puncture site, particularly after ACP implantation (*p* = 0.014). Significant peridevice leaks (PDL) were observed in 21.4% of simple sealing mechanism devices versus 0% in double sealing mechanism devices (*p* = 0.004). Thrombi were detected on devices in six patients, with no subsequent ischemic stroke or thromboembolic event. Comparative analysis revealed no significant differences in the occurrence of stroke, transient ischemic attack (TIA), thromboembolic events, device-related thrombi, or mortality among different device types. A 62.3% relative risk reduction in thromboembolic events and 38.6% in major bleedings could be observed over 568.2 patient years. Conclusions: In summary, our study highlights the efficacy and safety of LAA closure using various occluder devices despite anatomical challenges. Our long-term follow-up findings support LAA closure as a promising option for stroke prevention in selected patient cohorts. Further research is needed to refine patient selection criteria and optimize outcomes in LAA closure procedures.

## 1. Introduction

Percutaneous Left atrial appendage (LAA) closure is an increasingly popular intervention for the prevention of cardioembolic events in patients with nonvalvular atrial fibrillation, particularly for those who have contraindications to or are intolerant of oral anticoagulation therapy. Atrial fibrillation (AF) stands as the most prevalent sustained cardiac arrhythmia globally, affecting millions of individuals and substantially elevating the risk of ischemic stroke by up to fivefold. Within the realm of AF management, stroke prevention stands as a central objective, and LAA closure has emerged as a feasible non-pharmacological alternative for specific patient cohorts [1]. 

The LAA has been recognized as the principal source of thrombus formation in individuals with nonvalvular AF [2]. Over the last two decades, diverse occluder devices have been designed and enhanced to facilitate LAA closure procedures. These devices are specifically engineered to securely seal off the LAA, thereby averting the potential embolization of any formed thrombi [3]. The most widely used devices include the Watchman/FLX (Boston Scientific, Marlborough, MA, USA) and Amplatzer Amulet (Abbott, Santa Clara, CA, USA). Each device has its own distinctive design, mechanism of action, and procedural approach, factors that have the potential to influence the safety, effectiveness, and long-term consequences of LAA closure [4].

In this study, we conducted a comprehensive evaluation of four different occluder devices used for LAA closure: Watchman 2.5, Watchman FLX, Amplatzer Cardiac Plug (ACP), and Amulet. Our analysis encompassed a wide range of factors, including device-specific variables, patient demographics, clinical and echocardiographic parameters, LAA anatomical characteristics, laboratory data, peri- and post-interventional complications, as well as clinical endpoints such as stroke incidence, bleeding events, thrombi on devices, or death over the study duration. This comprehensive examination allows us to gain insights into the real-world performance of these devices, identify potential areas for enhancement in procedural techniques and device design, and serve as a valuable resource for guiding clinical decision-making and refining patient selection criteria for LAA closure procedures.

## 2. Materials and Methods

### 2.1. Study Design and Population

This retrospective monocentric cohort study included 270 patients who underwent LAA closure between 2009 and 2023 at our institution, University Clinic Heidelberg, Germany. Patients were eligible for an LAA closure if they had nonvalvular atrial fibrillation and were contraindicated for or intolerant of long-term oral anticoagulation therapy. The study protocol was approved by the institutional review board and local ethics committee, and the requirement for informed consent was waived due to the retrospective nature of the study.

### 2.2. Data Collection

We extracted patient data from electronic medical records, including demographics, clinical characteristics (AF type, CHA2DS2-VASc and HAS-BLED scores, etc.), LAA closure device type and size, laboratory values, LAA anatomy, peri- and postprocedural complications, electrocardiographic parameters, echocardiographic parameters from transthoracic and transesophageal echocardiography (TEE), and discharge medication. Moreover, fluoroscopy time and implantation duration, as well as post-implantation clinical events up to one year, were collected.

### 2.3. LAA Closure Procedure

The LAA closure procedures were performed by experienced interventional cardiologists, adhering to established techniques specific to the chosen devices. Before the intervention, all patients underwent TEE to evaluate LAA anatomy and verify the absence of thrombi. The selection of device type and size depended on LAA anatomy and the preferences of the operator. Fluoroscopy time during implantation, amount of contrast medium usage, and total procedure duration were documented. A follow-up TEE was scheduled at the three-month mark to assess device placement, identify any residual flow, and detect potential complications.

### 2.4. Outcome Measures

The main endpoints of the study were periprocedural success rate, absence of residual shunt during implantation and during follow-up evaluation, as well as periprocedural complication rate, including groin complications following the puncture, pericardial effusion, thrombus formation, stroke, device embolism, or LCX occlusion. Bleeding events were classified according to the Bleeding Academic Research Consortium (BARC) criteria [5]. Recurrent stroke was defined as a new-onset focal neurological deficit lasting more than 24 h with radiological confirmation. Further endpoints included bleeding, stroke rate, thromboembolisms, thrombi on devices, and death during follow-up evaluation.

### 2.5. Statistical Analysis

Continuous variables were expressed as mean ± standard deviation or median with an interquartile range, depending on the distribution of data. Categorical variables were presented as counts and percentages. Comparisons between groups were performed using the chi-square or Fisher’s exact test for categorical variables and the Kruskal–Wallis test for continuous variables. In the case of statistically significant differences, pairwise comparisons were performed using Dunn’s procedure. A two-sided *p*-value of <0.05 was considered statistically significant. Survival analyses for primary endpoints were conducted using the Kaplan–Meier method. All statistical analyses were performed using RStudio 2023.03.1+446 (R. Posit Software, PBC, Boston, MA, USA).

The relative risk reduction (RRR) in the annual occurrence of thromboembolic events (ischemic stroke and other thromboembolisms) and major bleedings was calculated by comparing the observed rates in this study to the expected rates based on the CHA2DS2-VASc and HAS-BLED scores.

## 3. Results

### 3.1. Patient Characteristics

The study included a total of 270 patients who underwent LAA closure from 2009 to 2023. Of these, 265 patients experienced successful implantation of occluder devices. Table 1 presents the basic characteristics of these successfully implanted patients, categorized by the type of device implanted. The mean age was 75.5 ± 7.7 years, and 64.5% were male. Almost all patients had AF (2 with atrial flutter), with the most common form being paroxysmal (48.3%). The median CHA2DS2-VASc score was 4.8 ± 1.5, and the median HAS-BLED score was 3.8 ± 1.0.

The most common indication for LAA closure was past bleeding events. Gastrointestinal and intracranial/intraspinal bleedings were the most frequent (38.5% each). Bleeding events of other origins were less frequent and included spontaneous intramuscular or intracutaneous bleeding in 13 patients (4.9%), severe epistaxis in 13 patients (4.9%), hematuria in 11 patients (4.2%), and ocular bleedings in 4 patients (1.5%). The second group of indications for LAA closure included individuals with an elevated risk of bleeding, yet without any previous bleeding incidents (Table 2).

### 3.2. Device Evolution

The procedural data indicated a distribution of device usage with a notable evolution over the study period. Initially, the Watchman 2.5 device was the most commonly employed (63.8%). However, with the introduction of the Watchman FLX, we observed a shift in device preference, which aligns with advancements in technology and changes in clinical guidelines. For instance, studies such as those performed by Galea et al. showed that Watchman FLX, as compared to Watchman 2.5, was associated with similar procedure-related complications and 6-month net adverse cardiovascular events (NACE), but with improved LAA neck coverage, and lower intradevice leaks and device-related thrombi (DRT) [6]. Also, the Amplatzer Cardiac Plug (ACP) device (10.6%), which was prevalent in the earlier years, saw a decline in use as newer devices such as the Amulet became available.

### 3.3. Procedural Outcomes and Complications

The overall procedural success rate was 95.7%. The initial success rates for implantation did not significantly differ between device types, being 94.9% for Watchman 2.5, 96.4% for Watchman FLX, 100.0% for ACP, and 95.2% for Amulet. From 13 initially unsuccessful device implantations, 1 could be implanted in the same procedure after changing device type and 7 could be successfully implanted in a second session. In five cases, no further attempts were made. No significant differences were found between the devices regarding fluoroscopy duration (12.0 ± 6.8 min), the average total procedure duration (62.5 ± 22.8 min), or the amount of contrast medium used (57.7 ± 37.7 mL). However, the total radiation dose differed significantly, with Watchman 2.5 being the highest (51.1 ± 38.0 Gy ×cm^2^) and Watchman FLX being the lowest (27.0 ± 23.5 Gy × cm^2^) (Table 3).

The atrial appendage anatomy in the cohort was distributed as follows: 26.5% chicken-wing, 28.3% windsock, 24.3% cactus, and 20.9% cauliflower. Cactus anatomy posed the most challenges across all devices regarding successful implantation. Fluoroscopy duration, average total procedure duration, amount of contrast medium used, and total radiation dose did not differ significantly between different atrial appendage anatomies (Supplementary Table S1). 

Periprocedural complications were defined as events up to discharge or up to seven days after implantation, based on previous studies like PREVAIL and the multicenter ACP study [7,8]. The most common periprocedural complication was bleeding over the puncture site, which was significantly more prevalent after ACP (*p* = 0.014). Seven (2.5%) bleeding cases were classified as BARC 3a and two (0.7%) as BARC 3b. Further periprocedural complications did not significantly differ between the devices and included pericardial effusion in six patients (2.2%), of which four had no clinical significance and did not require pericardiocentesis, transient ischemic attack (TIA) in one patient (0.6%) after Watchman 2.5 device implantation, manifesting as temporary aphasia with word-finding difficulties and slurred speech, and air embolism in one patient (0.4%) with a Watchman 2.5 device, leading to left-sided cerebral ischemia. No periprocedural device embolism or left circumflex artery (LCX) occlusion was reported (Table 4).

### 3.4. Peridevice Leak (PDL)

**Figure 1:**
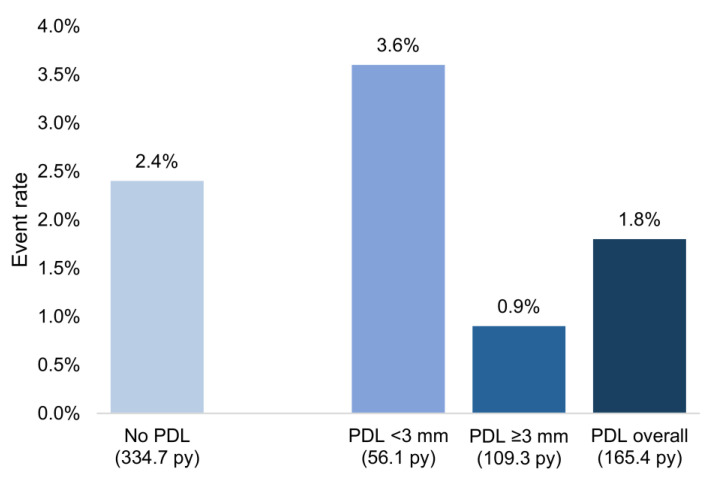
Rate of ischemic strokes and thromboembolisms per patient-year in relation to peridevice leaks (PDLs): Analysis demonstrates no significant increase in the incidence of ischemic strokes or thromboembolic events among patients with minor or major PDLs compared to those without leaks.

The incidence of significant PDL (≥3 mm) three months after implantation and the time at which the TEE was conducted varied significantly among devices with different sealing mechanisms. Devices with a simple sealing mechanism, such as Watchman/FLX, exhibited a higher leak incidence of 21.4%, whereas devices with a double sealing mechanism, like Amulet and ACP, demonstrated no significant PDL (*p* = 0.004); However, the rate of ischemic strokes and thromboembolisms per patient-year was not higher in patients with minor or major PDL (2.4% in patients without and 1.8% in patients with PDL) (Figure 1). The anticoagulation/antiplatelet strategies of patients with PDL are listed in Supplementary Table S2. 

### 3.5. Endpoints

**Figure 2:**
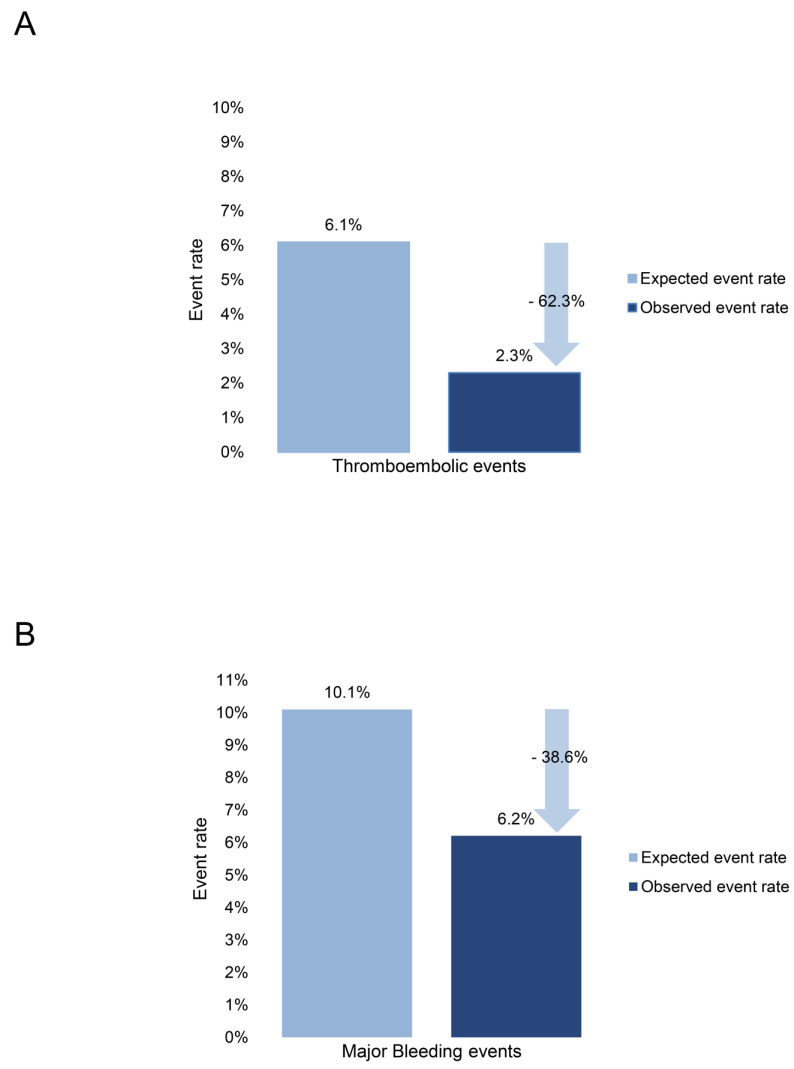
Relative risk reduction (RRR) over 568.2 patient years for thromboembolic events (**A**) and major bleedings (**B**).

After discharge and during the 12-month follow-up period, recurrent stroke or TIA occurred in four and thromboembolic events in one patient (pulmonary embolism). Thrombi were detected on devices in six patients who were subsequently placed on short- or long-term oral anticoagulation (OAC) or long-term aspirin (ASS) therapy. Up to the day of last contact, none of these patients had suffered an ischemic stroke or thromboembolic event. Three patients died during the follow-up period. The incidence of stroke, TIA, thromboembolic events, thrombi on devices, or death did not significantly differ between various device types (Table 5). The relative risk reduction (RRR) over the course of 568.2 patient years was 62.3% in the annual occurrence of thromboembolic events and 38.6% in major bleedings (Figure 2).

## 4. Discussion

This study’s comprehensive evaluation of percutaneous left atrial appendage (LAA) closure using various occluder devices at our center from 2009 to 2023 underscores the procedure’s efficacy and safety in patients with nonvalvular AF who are contraindicated for or intolerant to oral anticoagulation therapy. Continuous improvements in the LAAC procedure and device technology have significantly contributed to the procedural safety and efficacy, as evidenced by the consistent high success rates in major trials such as PROTECT (90.9%), CAP (94.4%), PREVAIL (95.1%), CAP2 (94.8%), and EWOLUTION (98.5%) [9]. The overall procedural success rate of 95.7% in our study showed no significant difference between devices, demonstrating the technical feasibility of LAA closure across different device generations. We further observed that the duration of fluoroscopy, total procedure time, and amount of contrast medium required were similar across devices, indicating a consistent level of technical performance and efficiency. 

The atrial appendage anatomy presents variable challenges in device implantation, with the cactus anatomy being the most challenging. However, LAA anatomy had no significant impact on procedural metrics in our study, suggesting that the success of LAA closure is more dependent on the operator’s experience and the technological features of the occluder devices than the anatomical classification alone.

Periprocedural complications, while relatively low, draw attention to the need for thorough procedural planning and performance as well as the necessity for careful postprocedural patient monitoring. The most common periprocedural complication was major bleeding over the puncture site, which was significantly more prevalent after ACP (*p* = 0.014). Further complication rates, including pericardial effusion, were consistent with rates reported in the literature [10,11]. Serious complications such as periprocedural device embolism or LCX occlusion were not observed in this study.

Whereas many studies, including ours, show no significant increased risk of stroke or thromboembolic events associated with PDLs, three studies have suggested that PDLs could indeed pose a risk for such adverse events, underscoring the need for further research [12,13,14]. In our study, the variability in PDL incidence was especially relevant between devices with simple versus complex sealing mechanisms, suggesting that device design may influence the risk of PDLs and their clinical significance. Korsholm et al. demonstrated that PDL at the disc was more common after Amulet device implantation compared to Watchman FLX. This difference might be attributable to the varying methods used for evaluating PDL or contrast patency (TEE in our study vs. cardiac CT in Korsholm et al.’s study) [15]. Our success rate, measured by the absence of PDL after Watchman/FLX implantation, was comparable to that reported by Korsholm et al. However, our study showed significantly better sealing results after ACP/Amulet implantation.

Thrombus formation on LAA occluders (DRT) is detectable by TEE and computed tomography and is reported with an incidence of 2–5% [16]. In our cohort, the incidence was 4.2%, aligning with the upper reported range and emphasizing the need for careful monitoring. Factors contributing to thrombosis risk include renal insufficiency, hypercoagulability, pericardial effusion, low left ventricular ejection fraction, deep device implantation, large device size, etc. [17,18]. Although device-related thrombosis increases stroke risk fourfold, the annual stroke rate remains low at 0.3% per 100 patient years [19]. Treatment with heparin or oral anticoagulants has shown a 95% success rate in thrombus resolution, highlighting their effectiveness [20,21]. Our six patients with device thrombi were placed on short- or long-term OAC or long-term aspirin therapy based on their renal function and overall bleeding or thromboembolic risk. None had suffered an ischemic stroke or thromboembolic event up to the day of last contact. 

Long-term follow-up revealed no significant differences in stroke, TIA, or thromboembolic events between occluder device types, underscoring the safety and efficacy of LAA closure across various devices. Relative risk reductions of 62.3% in the annual occurrence of thromboembolic events and 38.6% in major bleedings over the course of 568.2 patient years observed in our study underscore the substantial potential of LAA closure to improve patient outcomes, supporting findings from the PROTECT AF and PREVAIL trials [22,23]. The consistency of these benefits across a real-world population adds to the growing body of evidence supporting LAA closure as a viable alternative to long-term oral anticoagulation for stroke prevention in selected patient cohorts.

In addition to the well-known Watchman and Amplatzer devices, several other LAA occluders have been developed and used clinically. The PLAATO device, introduced in 2001 by Sievert, was the first-in-human percutaneous LAAC device featuring a self-expanding nitinol cage coated with non-thrombogenic polytetrafluoroethylene. Despite favorable clinical trials, it was withdrawn from the market in 2006. The Occlutech LAA Occluder, approved in 2016, is a self-expanding conical-shaped nitinol wire mesh designed for ease of implantation and enhanced sealing properties. The WaveCrest device, approved in 2013, features a single-lobe nitinol design with 20 anchoring points and polyurethane foam for enhanced sealing, making it suitable for smaller LAA anatomies. The Ultraseal LAA closure device, with its dual articulating joint and multidirectional mobility, could accommodate various LAA shapes and sizes effectively. Epicardial devices like the Lariat, which combines endocardial and epicardial approaches, have demonstrated effective LAA closure with a 95% success rate, though they are not FDA-approved specifically for stroke prevention. The Sierra Ligation System, an epicardial-only device, offers a simplified procedure without the need for transseptal puncture, enhancing its applicability in certain clinical scenarios. Future comparative studies should also include other clinically used devices to provide a comprehensive assessment of their performance, safety, and long-term outcomes relative to the more commonly used devices like the Watchman and Amplatzer series [24].

### Limitation

Limitations of our study include its retrospective design and its monocentric nature, which may affect the generalizability of the findings. Future multicenter, prospective, and larger studies are needed to validate our observations across broader populations and to further refine patient selection criteria for LAA closure.

## 5. Conclusions

In conclusion, our findings affirm the procedural success and safety of LAA closure across a spectrum of occluder devices and patient anatomies. The ongoing evolution of device technology and procedural techniques promises to further enhance the outcomes of this important therapeutic option by decreasing the bleeding rate, incidence of pericardial effusion, radiation exposure, peridevice leak, and device-related thrombi.

## Figures and Tables

**Table 1:** Implanted patients’ basic characteristics.

	Watchman 2.5 (n = 169)	Watchman FLX (n = 27)	ACP (n = 28)	Amulet (n = 41)	All (n = 265)	*p*-Value
Age, mean (SD), years	75.4 ± 8.1	77.1 ± 7.1	74.6 ± 6.3	75.8 ± 7.3	75.5 ± 7.7	0.58
Males, n (%)	104 (61.5)	15 (55.6)	18 (64.3)	34 (82.9)	171 (64.5)	0.05
BMI, mean (SD), kg/m^2^	26.9 ± 4.9	26.1 ± 4.1	26.1 ± 4.5	26.7 ± 3.2	26.7 ± 4.6	0.86
Atrial fibrillation/flutter						
Paroxysmal, n (%)	78 (46.2)	18 (66.7)	8 (28.6)	24 (58.5)	128 (48.3)	0.018
Persistent, n (%)	16 (9.5)	0	8 (28.6)	2 (4.9)	26 (9.8)	0.004
Long-standing persistent n, (%)	42 (24.9)	6 (22.2)	11 (39.3)	6 (14.6)	65 (24.5)	0.14
Atrial flutter, n (%)	2 (1.2)	0	0	0	2 (0.8)	1.00
CHA2DS2-VASc Score, mean (SD)	4.8 ± 1.5	5.1 ± 1.5	4.8 ± 1.3	4.5 ± 1.6	4.8 ± 1.5	0.36
HAS-BLED Score, mean (SD)	3.7 ± 1.0	3.9 ± 1.0	3.9 ± 0.7	3.6 ± 1.1	3.8 ± 1.0	0.52
Arterial hypertension, n (%)	161 (95.3)	26 (96.3)	27 (96.4)	37 (90.2)	251 (94.7)	0.61
Coronary artery disease, n (%)	107 (63.3)	17 (63.0)	10 (35.7)	22 (53.7)	156 (58.9)	0.042
ACI stenosis, n (%)	12 (7.1)	1 (3.7)	2 (7.1)	2 (4.9)	17 (6.4)	1.00
Diabetes mellitus, n (%)	54 (32.0)	10 (37.0)	7 (25.0)	15 (36.6)	86 (32.5)	0.73
Dyslipidemia, n (%)	95 (56.2)	19 (70.4)	10 (35.7)	25 (61.0)	149 (56.2)	0.06
History of syncope, n (%)	19 (11.2)	2 (7.4)	1 (3.6)	2 (4.9)	24 (9.1)	0.50
Peripheral arterial occlusive disease, n (%)	49 (29.0)	10 (37.0)	4 (14.3)	12 (29.3)	75 (28.3)	0.29
Smoking, n (%)	57 (33.7)	13 (48.1)	5 (17.9)	18 (43.9)	93 (35.1)	0.07
COPD, n (%)	18 (10.7)	4 (14.8)	1 (3.6)	4 (9.8)	27 (10.2)	0.60
Dyspnoea, n (%)						
NYHA ≤ II	128 (76.2)	24 (88.9)	26 (96.3)	35 (85.4)	213 (81.0)	0.042
NYHA > II	40 (23.8)	3 (11.1)	1 (3.7)	6 (14.6)	50 (19.0)	0.042
LV-EF (2D-TTE), mean (SD), %	51.5 ± 9.7	51.1 ± 9.9	NA	51.6 ± 6.0	51.4 ± 8.8	0.70
Chronic kidney disease	51 (30.2)	5 (18.5)	7 (25.0)	12 (29.3)	75 (28.3)	0.63
Serum creatinine, mean (SD), mg/dL	1.3 ± 1.0	1.0 ± 0.6	1.1 ± 0.4	1.4 ± 1.5	1.3 ± 1.1	0.36
hsTnT, median (IQR), pg/mL	16.0 (11.0–27.7)	22.3 (16.05–31.6)	17.0 (11.0–25.5)	50.0 (15.8–58.5)	17.45 (11.0–34.6)	0.026
NT-proBNP, median (IQR), ng/L	1231 (404–2523.7)	784 (531–1298)	1784 (1401–2101)	812 (356–2512.7)	1185.5 (390.2–2209.5)	0.630

BMI = body mass index. ACI = arteria carotis interna. COPD = chronic obstructive pulmonary disease. LV-EF = left ventricular ejection fraction. NYHA = New York Heart Association. hsTnT = high-sensitivity troponin T. IQR, interquartile range. NT-proBNP = N-terminal pro B-type natriuretic peptide.

**Table 2:** Indications for LAA closure.

	Watchman 2.5 (n = 169)	Watchman FLX (n = 27)	ACP (n = 28)	Amulet (n = 41)	All (n = 265)	*p*-Value
Past bleeding, n (%)						
Gastrointestinal	65 (38.5)	14 (51.9)	3 (10.7)	20 (48.8)	102 (38.5)	0.005
Intracranial/intraspinal	65 (38.5)	7 (25.9)	19 (67.9)	12 (29.3)	103 (38.5)	0.004
Spontaneous intramuscular/intracutaneous	5 (3.0)	3 (11.1)	4 (14.3)	1 (2.4)	13 (4.9)	0.024
Severe epistaxis	6 (3.6)	3 (11.1)	2 (7.1)	2 (4.9)	13 (4.9)	0.23
Hematuria	10 (5.9)	0	0	1 (2.4)	11 (4.2)	0.46
Ocular	3 (1.8)	0	1 (3.6)	0	4 (1.5)	0.67
Others	2 (1.2)	0	1 (3.6)	1 (2.4)	4 (1.5)	0.46
Elevated risk of bleeding, n (%)						
HAS-BLED ≥ 3	6 (3.6)	1 (3.7)	0	0	7 (2.6)	0.57
Pronounced propensity to fall	4 (2.4)	0	0	2 (4.9)	6 (2.3)	0.54
Hemophilia	1 (0.6)	0	0	2 (4.9)	3 (1.1)	0.17
Thrombocytopenia or compromised platelet function	2 (1.2)	0	0	0	2 (0.8)	1.00
Intracerebral cavernoma	0	0	0	1 (2.4)	1 (0.4)	0.37
Oral anticoagulant	2 (1.2)	0	2 (7.1)	1 (2.4)	5 (1.9)	0.15
Stroke despite OAC	4 (2.4)	0	0	1 (2.4)	5 (1.9)	1.00

One patient can have multiple primary indications simultaneously. OAC = oral anticoagulant.

**Table 3:** Procedural data in 277 LAAO implantation attempts.

	Watchman 2.5 (n = 178)	Watchman FLX (n = 28)	ACP (n = 28)	Amulet (n = 42)	All (n = 277 ^∞^)	*p*-Value
Successful implantation, n (%)	169 (94.9)	27 (96.4)	28 (100)	40 (95.2)	265 (95.7)	0.9
Failed Implantation, n (%)	8 (4.5)	1 (3.6)	0	2 (4.8)	12 (4.3)	0.9
Successful after intraprocedural device change, n (%)	1 (0.6)	NA	NA	NA	1 (0.4)	
Procedure duration, min	61.0 ± 22.2	59.6 ± 20.4	66.9 ± 21.3	67.7 ± 27.1	62.5 ± 22.8	0.3
Duration of fluoroscopy, min	11.5 ± 6.4	11.6 ± 5.4	14.4 ± 8.4	12.5 ± 8.1	12.0 ± 6.8	0.4
Radiation dose, Gy × cm^2^	51.1 ± 38.0	27.0 ± 23.5	49.6 ± 34.0	39.3 ± 44.0	46.6 ± 37.9	<0.0001
Amount of contrast medium, mL	59.7 ± 41.4	59.8 ± 33.5	45.9 ± 24.2	55.5 ± 30.3	57.7 ± 37.7	0.5
Sinus rhythm at admission, n (%)	73 (41.0)	12 (42.9)	6 (21.4)	18 (42.9)	109 (39.6)	0.2
Mean left atrial pressure at implantation, mmHg	19.3 ± 8.3	15.9 ± 5.3	17.2 ± 6.0	15.8 ± 9.0	18.1 ± 8.0	0.2
Hospital stay > 1 night, n (%)	59 (33.1)	7 (25.0)	17 (60.7)	6 (14.3)	90 (32.5)	0.0007

∞ = In one patient, an initial LAAO attempt had to be aborted due to hemodynamic instability before a device was selected. NA = not available.

**Table 4:** Periprocedural complications in 277 LAAO implantation attempts.

	Watchman 2.5 (n = 178)	Watchman FLX (n = 28)	ACP (n = 28)	Amulet (n = 42)	All (n = 277 ^∞^)	*p*-Value
Pericardial effusion, n (%)	5 (2.8)	0	0	1 (2.4)	6 (2.2)	1.0
Clinically relevant, n (%)	1 (0.6)	0	0	1 (2.4)	2 (0.7)	0.58
Pericardial tamponade, n (%)	0	0	0	1 (2.4)	1 (0.4)	0.35
Device embolism, n (%)	0	0	0	0	0	NA
LCX occlusion, n (%)	0	0	0	0	0	NA
TIA, n (%)	1 (0.6)	0	0	0	1 (0.4)	1.0
Myocardial infarction, n (%)	0	0	0	0	0	NA
Thromboembolism, n (%)	0	0	0	0	0	NA
Air embolism, n (%)	1 (0.6)	0	0	0	1 (0.4)	1.0
Death, n (%)	0	0	0	0	0	NA
Bleeding at puncture site, n (%)	19 (10.7)	0	4 (14.3)	0	24 (8.7)	0.014
Aneurysma spurium	7 (3.9)	0	2 (7.1)	0	9 (3.2)	0.27
BARC classification of bleeding						
Minor	13 (7.3)	0	2 (7.1)	0	15 (5.4)	0.14
1	0	0	0	0	0	
2	13 (7.3)	0	2 (7.1)	0	15 (5.4)	0.14
Major	6 (3.4)	0	2 (7.1)	0	9 (3.2)	0.31
3a	4 (2.2)	0	2 (7.1)	0	7 (2.5)	0.29
3b	2 (1.1)	0	0	0	2 (0.7)	1.0
3c	0	0	0	0	0	NA
4	0	0	0	0	0	NA
5a	0	0	0	0	0	NA
5b	0	0	0	0	0	NA
AV fistula without bleeding	3 (1.7)	0	0	0	3 (1.1)	1.0

∞ = In one patient, an initial LAAO attempt had to be aborted due to hemodynamic instability before a device was selected. LCX = left circumflex artery. TIA = transient ischemic attack. BARC = Bleeding Academic Research Consortium. NA = not available.

**Table 5:** Postprocedural events within one year of implantation.

	Watchman 2.5	Watchman FLX	ACP	Amulet	All	*p*-Value
Ischemic stroke, n (%)	2 (2.1)	0	0	1 (5.9)	3 (2.1)	0.481
TIA, n (%)	1 (1.0)	0	0	0	1 (0.7)	1.000
Thromboembolic events, n (%)	1 (1.0)	0	0	0	1 (0.7)	1.000
Thrombi on device, n (%)	2 (2.1)	1 (14.3)	2 (8.7)	1 (5.9)	6 (4.2)	0.116
Death, n (%)	3 (3.1)	0	0	0	3 (2.1)	1.000

TIA = transient ischemic attack.

## Data Availability

The original contributions presented in the study are included in the article.

## Supplementary Materials

The following supporting information can be downloaded at: https://www.mdpi.com/article/10.3390/jcdd11060158/s1, Table S1: Procedural data in 277 LAAO implantation attempts according to atrial appendage morphology; Table S2: Anticoagulation or antiplatelet strategies in patients with device-related thrombi.
