# Supplementary material for: Comparative Assessment of Percutaneous Left-Atrial Appendage Occlusion (LAAO) Devices—A Single Center Cohort Study
Source: J Cardiovasc Dev Dis. 2024 May 21;11(6):158. doi: 10.3390/jcdd11060158 (PMC11203807; doi:10.3390/jcdd11060158)
Supplement: Supplementary file 1 [file jcdd-11-00158-s001.zip › jcdd-2986004-supplementary.pdf]

Suppl. Table S1: Procedural data in 277 LAAO implantation attempts according to atrial appendage morphology

	**Chicken-Wing** (n = 71)	**Windsock** (n = 78)	**Cactus** (n = 68)	**Cauliflower** (n = 58)	**All** (n = 277)	**p-value**
Successful implantation	71 (100.0)	73 (93.6)	63 (92.6)	56 (96.6)	265 (95.7)	0.08
Watchman 2.5 (n = 178)	48	45	49	36	178	
Of these, successful	48 (100.0)	40 (88.9)	46 (93.9)	36 (100.0)	170 (95.5)	0.02
Watchman FLX (n = 28)	8	8	5	7	28	
Of these, successful	8 (100.0)	8 (100.0)	4 (80.0)	7 (100.0)	27 (96.4)	0.18
ACP (n = 28)	5	14	3	4	28	
Of these, successful	5 (100.0)	14 (100.0)	3 (100.0)	4 (100.0)	28 (100.0)	NA
Amulet (n = 42)	10	11	11	10	42	
Of these, successful	10 (100.0)	11 (100.0)	10 (90.9)	9 (90.0)	40 (95.2)	0.86
Procedure duration, min	62.7 ± 24.8	62.7 ± 22.3	61.4 ± 19.7	62.5 ± 24.5	62.5 ± 22.8	0.99
Duration of fluoroscopy, min	11.5 ± 7.8	11.9 ± 5.7	12.0 ± *6.7*	12.6 ± 7.1	12.0 ± 6.8	0.51
Radiation dose, Gy*cm^2	39.4 ± 33.0	46.6 ± 37.4	52.8 ± 40.2	47.7 ± 41.4	46.6 ± 37.9	0.15
Amount of contrast medium, ml	57.2 ± 39.2	52.5 ± 33.8	55.2 ± 32.4	67.6 ± 45.1	57.7 ± 37.7	0.23

Suppl. Table S2: Anticoagulation or antiplatelet strategies in patients with device-related thrombi.

	Anticoagulation/ antiplatelet strategy	Duration of this medication	DRT detected at	Anticoagulation/ antiplatelet medication at time of detection	Consequence	Development of DRT
**ACP**	ASS100, Clopidogrel 75	10 days; then Clopidogrel 75 only (due to M. Osler related bleeding anaemia)	1 month post implantation	Clopidogrel 75	Start of temporary LMWH	18 days after first DRT detection and beginning of LMWH treatment, DRT still visible. LMWH dosage was raised till the last contact.
**ACP**	ASS100, Clopidogrel 75	Until detection of DRT	1 month post implantation	ASS100, Clopidogrel 75	Start of NOAC (Xarelto)	Several TEE controls with visible DRT. Consequence: 15 mg Xarelto lifelong.
**Watchman 2.5**	ASS100, Clopidogrel 75	Until detection of DRT	1 month post implantation	ASS100, Clopidogrel 75	Start of temporary NOAC (Xarelto)	Complete regression of the DRT. Followed by change from Xarelto to ASS100 only.
**Watchman 2.5**	ASS100, Clopidogrel 75	3 months; then ASS100 only	7 months post implantation	ASS100	Continuation of ASS (due to bleeding history)	Regression of the DRT.
**FLX**	ASS100, Clopidogrel 75	3 months; then ASS100 only	3.5 months post implantation	ASS100	Continuation of ASS (due to bleeding history)	Loss of contact to the patient after 1st detection of DRT.
**Amulet**	ASS100, Clopidogrel 75	3 months; then ASS100 only	12 months post implantation	ASS100	Start of temporary NOAC (Eliquis)	Regression of the DRT.

Medication dosis are in mg/d.
